# A novel carbon-fibre adjustable reusable accessory (CARA) for supine breast positioning to reduce toxicity in breast adjuvant radiotherapy: a study protocol for a multicentre phase III randomized controlled trial

**DOI:** 10.1186/s12885-022-09759-y

**Published:** 2022-06-20

**Authors:** Cheryl Duzenli, Elisa K. Chan, Alanah M. Bergman, Sheri Grahame, Joel Singer, Levi Burns, Robert A. Olson

**Affiliations:** 1 Department of Medical Physics, BC Cancer, Vancouver, BC Canada; 2 Department of Surgery, Division of Radiation Oncology and Developmental Radiotherapeutics, University of British Columbia, Vancouver, BC Canada; 3 Department of Physics and Astronomy, University of British Columbia, Vancouver, BC Canada; 4 Department of Radiation Oncology, BC Cancer, Vancouver, BC Canada; 5 Department of Radiation Therapy, BC Cancer, Vancouver, BC Canada; 6 School of Population and Public Health, University of British Columbia, Vancouver, BC Canada; 7 Centre for Health Evaluation and Outcome Sciences, St. Paul’s Hospital, Vancouver, BC Canada; 8 Michael G. DeGroote School of Medicine, McMaster University, Hamilton, ON Canada; 9 Department of Radiation Oncology, BC Cancer – Centre for the North, Prince George, BC Canada

**Keywords:** Breast cancer, Supine positioning, Adjuvant radiotherapy, Skin toxicity, Moist desquamation, Infra-mammary fold, Breast support, Quality of life, Patient-reported outcomes

## Abstract

**Background:**

A novel device for supine positioning in breast radiotherapy for patients with large or pendulous breasts has been developed and tested in phase II studies. This trial is designed to assess the efficacy of the device to reduce skin toxicity and unwanted normal tissue dose in comparison to the current clinical standard for supine breast support during breast radiotherapy.

**Methods:**

Patients at high risk for moist desquamation, having infra-mammary fold or lateral ptosis, will be randomized into two arms. Patients in the control arm will receive breast radiotherapy with supine positioning using current standard of care. Patients in the experimental arm will be positioned supine with the novel device. The primary endpoint is the incidence of moist desquamation in the infra-mammary fold. We hypothesize a 20% reduction (from 50 to 30%) in the rate of moist desquamation in the study arm versus the control arm. For 80% power, two-tailed α = 0.05 and 10% loss to follow up, 110 patients will be assigned to each arm. The proportion of patients experiencing moist desquamation in the two arms will be compared using logistic regression adjusting for brassiere cup size, skin fold size, body mass index, smoking status, and dose fractionation schedule. An unadjusted comparison will also be made using the chi-square test, or Fisher’s exact test, if appropriate. Secondary endpoints include dose-volume statistics for the lung and heart, skin dose and clinical parameters including setup time, reproducibility, and staff experience with setup procedures. Patient-reported pain, skin condition interference with sleep and daily activities, and comfort during treatment are also secondary endpoints.

**Discussion:**

Based on results from earlier phase II studies, it is expected that the device-enabled elimination of infra-mammary fold should reduce toxicity and improve quality of life for this patient population. Earlier studies showed reduction in dose to organs at risk including lung and heart, indicating potential for other long-term benefits for patients using the device. This study is limited to acute skin toxicity, patient-reported outcomes, and clinical factors to inform integration of the device into standard breast radiotherapy procedures.

**Trial registration:**

Clinicaltrials.gov identifier: NCT04257396. Registered February 6 2020.

**Supplementary Information:**

The online version contains supplementary material available at 10.1186/s12885-022-09759-y.

## Background

The majority of patients with in-situ and early-stage breast cancer receive adjuvant breast radiation therapy (RT) after breast conserving surgery, with adjuvant RT reducing the relative risk of cancer recurrence by half to two thirds [1] while offering minimal overall toxicity, good cosmetic results, and improved quality of life [2].

Some breast RT patients experience skin toxicity [3], which may include moist desquamation (MD), a painful breakdown of skin that can impact quality of life [4]. Development of MD has been found to depend on multiple factors, including the skin dose, the presence of skin folds, breast size, breast ptosis, and body mass index (BMI). Several studies of supine breast RT using varying fractionation schedules, use of beam-modifying wedges or intensity-modulated radiation therapy (IMRT), all concur that the group expected to be at highest risk of MD consists of patients having prominent infra-mammary fold (IMF) areas, BMI greater than 30, brassiere cup size D or larger, and large mid-breast separation [5–8].

Various approaches to supine breast positioning for patients with large or pendulous breasts have been investigated [9]. Currently, there is no accepted standard for supine breast positioning supported by peer-reviewed evidence for large or pendulous breasts. Brassiere use in supine breast RT was reported to reduce lung and heart volume for left breast patients but resulted in higher rates of acute skin toxicity [10]. Thermoplastic shells have been reported to reduce heart dose for left breast patients who are not eligible for breath hold techniques, but this technique is problematic for very large patients and rate of occurrence and degree of MD has not been reported [11]. Prone positioning has also been investigated as a potential solution to the problem of skin folds for pendulous breasts [12–20]. While some success has been achieved in reducing lung dose and eliminating IMF, the prone technique is generally not suitable for patients requiring nodal irradiation and may increase dose to the heart in some treatment conditions. Moreover, the prone position is not tolerable for all patients. The risk of MD in the infra-mammary area for the large or pendulous breast population is expected to be up to 50% with standard supine positioning, even with IMRT, when the whole breast is treated.

Interpretation of study results with respect to MD is made challenging by the fact that in standard scoring systems, MD appears in both grades 2 and 3 (e.g., Radiation Therapy Oncology Group (RTOG) and National Cancer Institute – Common Terminology Criteria for Adverse Events (NCI CTCAE V5.0)). In addition, many studies do not document BMI or the presence of skin folds, and many studies do not monitor skin toxicity out to two weeks post-treatment when at least 10% of skin reactions peak [8] for standard fractionation schedules, which can result in under-reporting of MD.

In breast RT, skin dose depends on factors including the planning technique, dose constraints, dose prescription, and beam energy. Patients having skin folds during treatment are expected to develop MD in the fold because delivering the prescribed breast dose involves the skin fold dose reaching over 95% of the prescription dose, as it lies within the treatment volume. The risk of MD has also been linked with the volume of the treated breast receiving 105% or more of the prescribed dose (breast V105%) [21–23].

**Fig. 1:**
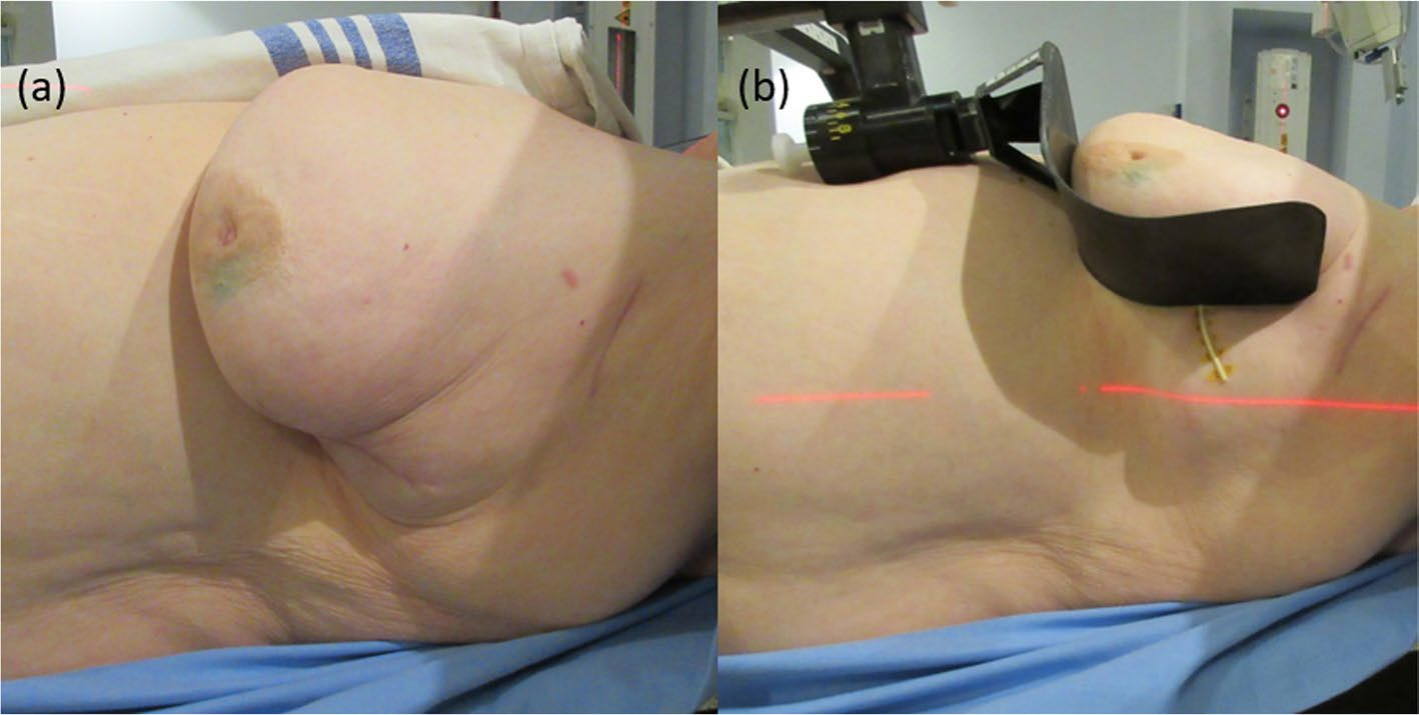
Patient without CARA support (**a**) and with CARA support (**b**). The CARA carbon fibre cradle supporting the breast is visible

**Fig. 2:**
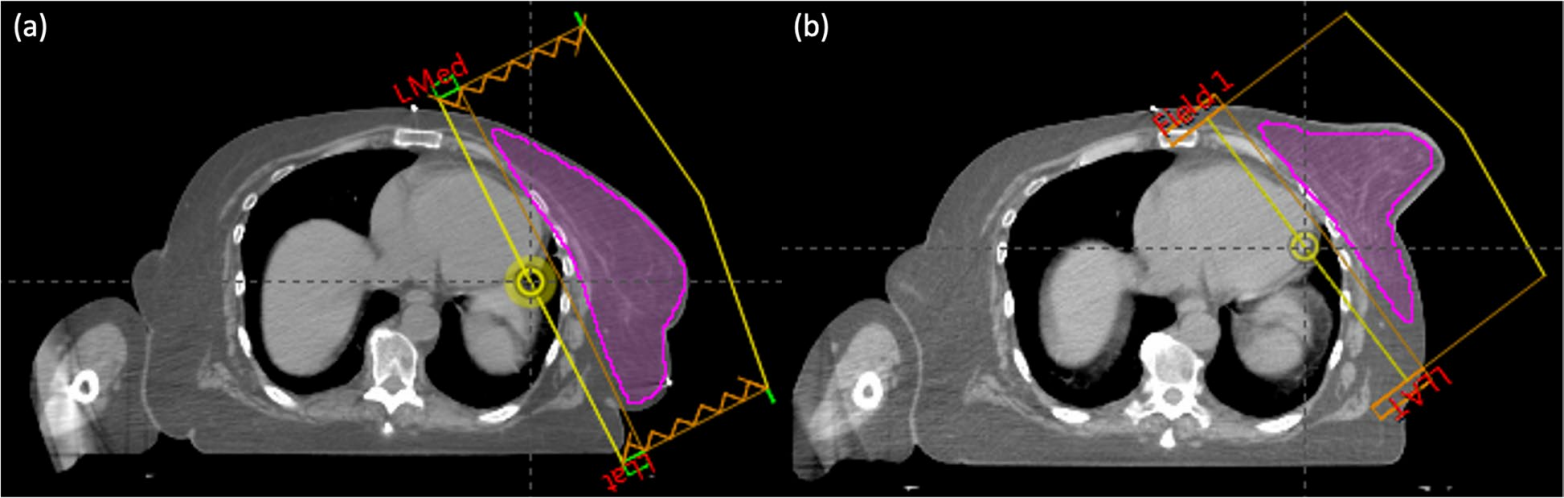
Axial CT images of the patient shown in Fig. 1 without CARA (**a**) and with CARA (**b**). The tangential beams and target volume are shown

To reduce MD incidence in this patient population, a novel Carbon-fibre Adjustable Reusable Accessory (CARA) for breast support was designed, fabricated, and tested beginning in 2016 [24, 25]. The current CARA clinical prototype is shown in use on a patient in Fig. 1. In a pilot phase II trial using this first clinical prototype [26], 30 patients at highest risk for MD based on BMI, skin folds, and breast cup size underwent computed tomography (CT) simulation and treatment planning both with and without CARA. The first 10 patients were treated with current standard of care and the last 20 patients were treated using CARA V1.0. The clinical treatment planning volume dose constraint for the step and shoot (field-in-field) forward-planned IMRT technique for large breasts at the time was V107% < 20 cm3. There was no constraint on V105%. CT images of the patient in Fig. 1 are shown in Fig. 2 with and without CARA. Dose-volume statistics demonstrated a systematic reduction in medial–lateral breast separation and associated reductions in ipsilateral lung dose volume receiving 20 Gy or more (lung V20Gy, *p* = 0.005), volume of breast receiving 105% or more of the prescribed dose (breast V105%, *p* = 0.006) and volume of body treated (body V50%, *p* = 0.001) without compromising target coverage. All IMFs in this study were eliminated on CARA treatment plans. Peak skin reactions for the CARA treated group were 10% grade 1, 75% grade 2 and 15% grade 3 using CTCAE V4.0, and daily variation in treatment setup was consistent with standard supine positioning [26].

Following the phase II study, three significant changes were implemented to reduce the incidence of MD in the context of IMF removal using CARA. First, the CARA cradle was re-designed to remove dose due to scatter arising from the attachment to the supporting bracket, resulting in CARA V2.0. Second, the material system was re-designed to reduce the areal density of the entire carbon-fibre cradle by 50%, further reducing the expected skin dose by approximately 10% across all areas of the breast in contact with the cradle. The current version of the cradle, CARA V3.0, incorporates this modification. In addition, a sliding window IMRT technique has now been introduced for tangential breast irradiation. With the established benefits of removing the IMF and reducing breast separation using CARA, tighter dose constraints on V105% can now be met using the sliding window technique. An IMRT dose constraint of V105% < 2% of the optimization target volume (OTV) is now in use.

To address deficiencies in measuring MD using standard scoring systems [27] and to alleviate the difficulty in having patients return to the clinic multiple times post-RT, patient-reported outcomes (PRO) are now the primary measure. Patient-reported outcomes are recognized as important clinically relevant outcome measures in modern radiation oncology [28–31]. Our institution established and validated the Prospective Outcomes and Support Initiative (POSI) methodology to collect PRO information [32]. A recent study investigated PROs for degree and location of skin reaction, pain, sleep disruption, and activity interruption for patients receiving whole breast RT [33]. The sensitivity and specificity for patient-reported incidence of MD were 87% and 76% respectively using open skin as an indicator of MD. The PRO instrument used in that study is in use in this trial.

Having demonstrated the potential clinical benefit of CARA through reduction in doses to organs at risk (OARs) [26], the current study is designed to assess the effectiveness of utilizing CARA V3.0 to reduce the incidence of acute skin reactions in the IMF in patients at high risk of MD undergoing breast RT. In this protocol, the IMF refers to the areas of skin on the inferior breast surface and on the chest that are in contact when the breast is unsupported in the supine position. The IMF is effectively eliminated when using CARA, but skin reactions are being monitored on this skin area after removal of the breast support, post-RT delivery. Compared to the pilot phase II trial, this is a phase III randomized controlled trial (RCT) on a larger population that incorporates improvements from the pilot and the use of PROs for the primary outcome measure. In addition, a discussion of the clinical acceptability of CARA, with respect to workflow measures, setup reproducibility, and therapist satisfaction, will be provided.

## Methods/Design

### Objectives

The primary objective of this study is to determine whether CARA V3.0 can reduce the incidence of moist desquamation in the IMF for patients with large or pendulous breasts undergoing breast RT in the supine position.

The secondary objectives of the study are as follows:

- To assess whether the severity of MD in the IMF can be reduced using CARA V3.0.
- To assess whether overall breast skin reaction scores can be reduced using CARA V3.0.
- To assess if CARA V3.0 reduces the volume of ipsilateral lung receiving 20 Gy or more (V20Gy).
- To assess if CARA V3.0 can reduce or maintain the volume of heart receiving 25 Gy or more (V25Gy) for left breast patients.
- To assess if CARA V3.0 can reduce the volume of body receiving greater than 50% (V50%) and greater than 105% (V105%) of the prescribed dose.
- To determine a dose-area parameter that is predictive of MD.
- To compare workflow and breast position reproducibility between the two study arms.
- To compare the PROs for quality of life measures and comfort during treatment setup between the two study arms.
- To compare radiation therapist experience between the two study arms.

### Study design

**Fig. 3:**
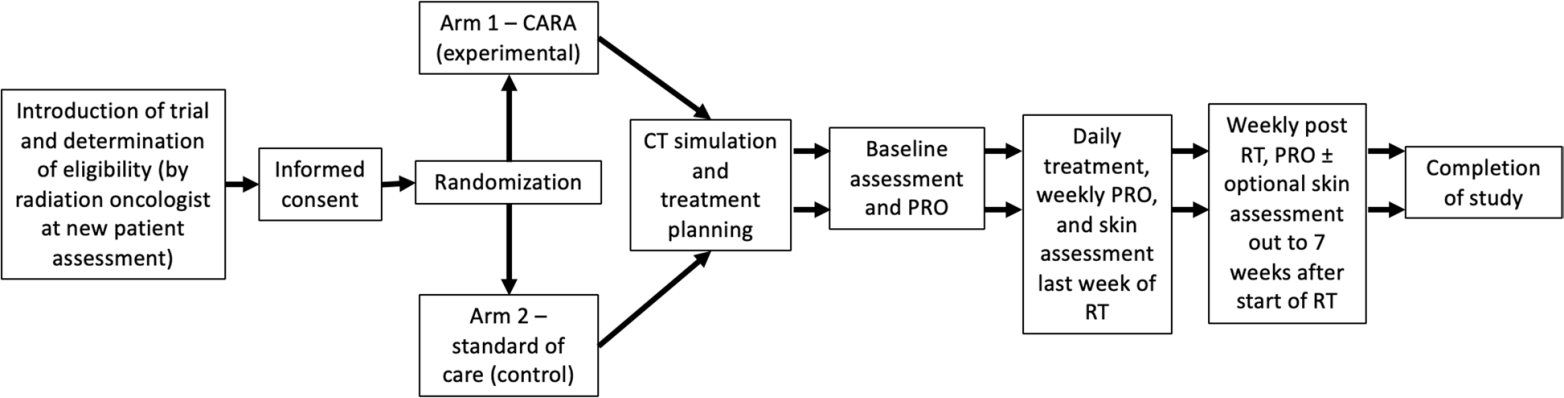
Study schema for each participant from identification and eligibility screening to study completion

This is a phase III multicentre trial with patients randomized to two treatment arms of equal sample size. Arm 1 (experimental arm) patients will receive CARA V3.0, while arm 2 (control arm) patients will be treated according to the current standard of care at the treatment centre (without CARA V3.0). The study schema is shown in Fig. 3.

### Primary endpoint and measures

#### Presence or absence of MD in the IMF

- Based on the PRO questionnaire (described under *Data Collection* [see Additional file 1]), a positive score of MD in the IMF will be based on a response of “Yes” for open skin within the past seven days (Question 7a) and a non-zero response for “under the breast” as a location of open skin (Question 7b), unless MD is definitively ruled out by a trained and blinded staff assessor.

### Secondary endpoints and measures

#### Degree of MD in the IMF (patchy vs. confluent and area of MD)

- Dimensions of any MD will be measured by using a ruler on the skin by trained staff blind to the treatment arm.
- A patch of MD is defined as a single spot up to 2 cm in its largest dimension. When patches exist, the dimensions of the overall patchy area and the dimensions of the largest patch will be recorded.
- When multiple patches have become confluent or a single patch is greater than 2 cm in its longest dimension, these areas will defined as confluent and the dimensions will be recorded.

#### Overall NCI CTCAE breast skin assessment

- The overall breast skin assessment will be scored using the NCI CTCAE V4.0 criteria for radiation dermatitis by the blinded study radiation therapist.

#### Treatment planning dose-volume statistics

- The following dose-volume measurements will be acquired:
  - ◦ V20 Gy ipsilateral lung
  - ◦ V25 Gy heart
  - ◦ V105% body
  - ◦ V50% body
  - ◦ Breast separation (cm)
- The measurements will be reported as relative volume (% of structure volume), absolute volume (cm^3^), relative dose (% of prescribed dose), and absolute dose (Gy).
- Breast separation (cm) will be measured from beam entry to beam exit at the posterior beam edge at mid-breast level.

#### Dose-area statistics measured *in-vivo* on breast skin

- Skin dose will be measured using GafChromic™ film in contact with the skin surface and analyzed using FilmQAPro™ software. Film dosimetry will be performed at the central coordinating centre on one to three treatment fractions depending on the prescribed dose fractionation schedule.
- In the experimental arm, the film will line the CARA support in contact with the breast.
- In the control arm, when achievable, the film will be placed along the chest wall with the superior edge of the film along the infra-mammary breast crease. This will place the film in the skin fold when a skin fold is present.

#### Patient-reported outcomes

- The PRO questionnaire will be provided on tablets in the clinic by the study radiation therapist on the days that the patient is being assessed and by e-mail post-RT.
- Secondary patient-reported outcomes include severity of fatigue, pain, sleep interruption, activity interruption, comfort during the treatment procedure, and use of steroid cream or analgesics.

#### Clinical workflow

- Simulations and treatments will be timed as workflow measures. These times will be measured by the study therapist from the point at which the patient enters the simulation or treatment room for the procedure until the patient exits the room post procedure.

#### Breast position reproducibility

- The position (Cartesian coordinates relative to a reference point) of anatomical features and surgical clip positions on kV imaging, MV imaging and cone-beam CT (CBCT) images taken at treatment will be compared.

#### Therapist experience

- Therapist experience with the treatment setup procedure will be collected using a purpose-built questionnaire for this study [see Additional file 2] to be completed by one or more therapists during the last week of treatment for each patient.

### Patient population

This study is designed for patients having large or pendulous breasts resulting in IMF and/or lateral breast ptosis. The oncologist or radiation therapist will use an eligibility checklist to pre-screen for potential participants. Patients receiving nodal RT are eligible assuming the inclusion criteria are met. Enrolment in other studies at the same time is permitted at the discretion of the treating radiation oncologist. Waivers to eligibility criteria are not permitted.

Patient participation will be required for seven weeks after the start of breast RT with PRO questionnaires to be completed weekly, starting the last week of treatment and through to the duration of this seven-week period. Follow-up assessments with staff may be done in the clinic or remotely (e.g. Tele-Health, Zoom, phone, e-mail).

#### Inclusion criteria

- Age 18 years or older
- Diagnosed with completely excised breast cancer (stage 0–3) or ductal carcinoma in situ (DCIS)
- Have undergone breast-conserving surgery and are scheduled to undergo adjuvant breast RT
- Able to provide informed consent (may involve a qualified interpreter for spoken language other than English)
- Eastern Cooperative Oncology Group (ECOG) performance status 0–2
- Any infra-mammary skin fold of 0.5 cm or larger and/or palpable lateral breast tissue falling posterior to the mid-axilla line with the breast unsupported in the supine treatment position
- Ability to complete skin self-assessments by PRO questionnaire

#### Exclusion criteria

- Inability to comply with experimental arm of trial
- Previous RT to either breast or chest
- Planned boost to infra mammary area (patients undergoing boost RT are otherwise eligible, assuming inclusion criteria are met)
- Planned partial breast irradiation that does not include the infra-mammary region
- Use of Mepitel® or other wound contact layer product while on treatment
- Failure to heal surgical wound or significant post-operative wound infection
- Presence of significant connective tissue disease (e.g., systemic sclerosis, systemic lupus erythematosus)
- Known radiation hypersensitivity phenotype (e.g., ataxia, telangiectasia)
- Inability to complete PRO questionnaire (including skin self-assessments) or to be assessed remotely out to 7 weeks after the start of breast RT

### Interventions

#### Radiation therapy

For both study arms, breast RT protocols currently in use at the treating centres will be used. Dose fractionation schedules include 26 Gy in 5 daily fractions, 42.5 Gy in 16 daily fractions, 40 Gy in 15 daily fractions, 45 Gy in 25 daily fractions, and 50 Gy in 25 or 28 daily fractions. The extended fractionation schedules are generally reserved for patients with a large separation (> 24 cm) or significant post-operative changes. Partial breast treatments are acceptable if the inferior breast skin is included in the treated volume.

The treatment delivery will generally consist of a tangent pair of photon fields with or without internal mammary, supraclavicular, and/or axillary nodal fields. Combinations of 6 MV, 10 MV, 15 MV or 18 MV photons are permitted as required to meet planning dose constraints. Volumetric modulated arc therapy (VMAT) is currently under consideration.

Additional boost radiation to the tumour bed may be prescribed at the discretion of the radiation oncologist. Boost may be delivered using MV photons or electrons before, during, or following breast RT.

In both arms, patients will be planned and treated in the supine position on an angled breast board. In addition, patients must be indexed to the breast board and the breast board must be indexed to the treatment couch. Deep inspiration breath hold (DIBH) for left breast patients may be used to reduce dose to the heart [34] in both study arms. Standard skin care instructions will be utilized.

#### CT simulation

CT simulation will be performed with the patient in supine position for both study arms. The preferred patient position is both arms above the head with elbows resting comfortably on a wing board. Variation in arm position (one arm up) is acceptable if the patient is unable to achieve both arms up. Arm position shall be documented on the treatment planning data sheet. Simulation will be performed using the standard CT protocol at the treating centre, confirmed during the credentialing review. Setup marks will be placed laterally and medially, and tattoos will be applied for future setup at the treatment unit. The preferred method for eliminating patient roll at the time of treatment is to place lateral tattoos on each side of the patient with arms up at the time of simulation. Alternate methods will be confirmed at the time of the credentialing review.

Detailed technical descriptions and figures of CARA are published elsewhere and similar to the completed pilot studies [24–26]. In the experimental arm, CARA settings will be adjusted on a patient-by-patient basis to eliminate the IMF and lateral tissue droop such that that all palpable breast tissue sits anterior to the mid-axilla line. Patient-specific CARA settings will be captured to include in the setup instructions at the treatment unit.

Patients in the control arm will be positioned according to standard practice of care which may include no support, a small foam wedge in the skin fold, a thermoplastic shell, or an alternative supine breast support approved at the time of credentialing review. The method will be recorded with the treatment planning data. No changes to standard practice in the control arm, as reported on the credentialing survey, are permitted during this study without consultation with the steering committee.

#### Target volumes and organs at risk

The breast clinical target volume (CTV) includes the palpable breast tissue and glandular and fatty breast tissue visualized by CT. The planning target volume (PTV) includes a 2 cm beam margin superior and inferior to the CTV and extends to the chest wall-lung interface but does not include skin unless clinically necessary. Supraclavicular, axillary, and internal mammary nodal PTVs will be identified and contoured according to current standard practice at the treating center. Up to 5 surgical clips within the breast PTV will be contoured when available, for positional measurement during imaging at the treatment unit.

An optimization target volume (OTV) is derived during the treatment planning process from a specified isodose surface (typically 30–50% of the prescribed dose) for open fields at the chosen lateral and medial beam angles, including any required blocking, as specified by the radiation oncologist. This isodose contour is cropped 5 mm in from the skin and lung and then labelled as OTV or Breast_Eval. Other approaches to target volume segmentation may be approved at the credentialing review.

The whole body and OARs will be contoured according to RTOG Atlas definitions. OARs include heart, ipsilateral lung, and any other OARs used in standard practice at the treating center. To mitigate difficulties with contouring, the maximum heart dose will serve as a temporary surrogate to the left anterior descending artery (LAD) dose for left breast cases. Future, detailed LAD dose analysis may be performed.

#### Treatment planning and dose constraints

Treatment planning will be performed for both study arms according to current standard of care using the Varian Eclipse AAA algorithm (V13.6) or equivalent, as assessed at credentialing or during the trial if system upgrades occur. Correction for lung inhomogeneity will be used.

PTV, OTV or Breast_Eval coverage by the 95% isodose surface is required. The dose homogeneity objective is V105% < 2% of the OTV. Beam energy may be adjusted as required to achieve PTV coverage and homogeneity criteria, however PTV coverage will take priority over dose homogeneity if the latter cannot be met. For treatments of 15, 16, 25, or 28 fractions, ipsilateral lung V20 Gy shall be ≤ 15% of the lung volume (≤ 35% if regional nodes are treated) and V25 Gy heart shall be ≤ 5% of the heart volume. For 26 Gy in 5 fraction treatments, ipsilateral lung V8 Gy shall be ≤ 15% of the ipsilateral lung volume, heart V1.5 Gy shall be ≤ 30% and heart V7 Gy shall be ≤ 5% of the heart volume. Sliding window or field-in-field (step and shoot) IMRT may be used as available at the treating center.

All treatment plans in this study will be approved by the treating oncologists and designated physicists at each site, including a quality assurance check on all contours. All constraint violations will be noted on the planning data form.

#### Treatment verification

Body and breast position during treatment will be verified with standard of care electronic portal imaging or kV imaging. Additionally, weekly kV CBCT will be performed, when achievable, to monitor the position of segmented surgical clips (when present).

### Data collection

#### Baseline assessment

Data collection activities are shown in Table 1. Patients will have a baseline assessment prior to the first treatment. Patient height and weight, BMI, age, smoking status, RT prescription (including boost), adjuvant chemotherapy, brassiere size (band and cup size), IMF skin fold depth in treatment position, and presence of other skin folds will be recorded. The skin will also be assessed at baseline for any pre-existing conditions such as erythema, telangiectasia, desquamation, or infection. At CT simulation, a baseline photograph of the IMF skin will be acquired as well as a photo of the patient in treatment position. A PRO questionnaire is also collected at baseline.

**Table 1:** Schedule of activities and data collection

**Study Activity**	**New patient appointment with radiation oncologist**	**Prior to CT simulation**	**Prior to first treatment**	**Weekly during Breast RT**	**Post-RT weekly to 7 weeks from start of breast RT**
Introduction to study	**X**				
Inclusion/exclusion criteria screening	**X**	**X**			
Informed consent obtained		**X**			
Enrollment and randomization		**X**			
Baseline assessment			**X**		
Patient-reported outcome questionnaire			**X**	**X**	**X**
Skin assessment performed by staff			**X**	**Last week of RT, or for five-day fractionation, last day of RT**	**Optional per patient preference**
Skin dose measurement				**Up to three times per patient; optional for sites other than the coordinating site**	
Timing of clinical flow				**X**	
Standard imaging				**X**	
CBCT imaging				**Up to three times per patient**	
Therapist satisfaction				**Last week of RT, or for five-day fractionation, last day of RT**	

#### Patient-reported outcomes

PRO shall be collected using the POSI methodology [32, 33]. The PRO questionnaire items are summarized above (section *Secondary Endpoints*) [see Additional file 1]. Patients who do not complete the PRO questionnaire on schedule will be contacted by the trial staff to follow-up.

The questionnaire will be available using tablets available in the clinic and by electronic copy post-RT. Alternatively, a physical copy can be delivered to the patient by the trials team, or it can be completed remotely with the assistance of the trials staff if the staff member is blinded to the treatment arm. The PRO questionnaire is currently available in English, Spanish, French, Hindi, Punjabi, and Chinese. Other translations can be made available upon request.

#### Staff skin assessments

Staff radiation therapists, nurses, or radiation oncologists blinded to the treatment arm will perform skin assessments. Staff performing these assessments are required to undergo specific training for this purpose. An in-house MD scoring tool specifically for the IMF will be used in addition to CTCAE V4.0 skin toxicity criteria. Staff skin assessments are required at baseline and during the last week of treatment (or in five-fraction prescriptions, required on the last day of treatment). If the patient is able and willing, staff skin assessments will also be completed weekly thereafter, out to 7 weeks after the start of breast RT.

Each staff skin assessment includes taking photographs of the treated skin in the IMF. Photos of other areas of skin experiencing MD at the time of each assessment may also be acquired. For patients assessed remotely, the patient may be asked to provide photographs of their skin taken at the time of the assessment.

While the method of breast positioning (i.e., Arm 1 or Arm 2) will be open label to the patient and treating staff, it is preferred that staff performing the follow-up skin assessments be blind to which arm of the study participants are in and not have access to the randomization allocation data. If this is not possible (such as in smaller centres), one additional trained study member will perform blinded assessment using photographs taken of the treated IMF area. If there is disagreement between the two assessments, a third trained study member will perform blinded assessment using the photographs. The final assessment of the primary outcome will be by majority vote.

#### *In-vivo* skin dosimetry

GafChromic™ film will be used to monitor skin dose on an around the infra-mammary breast skin during treatment on three fractions for treatment regiments ≥ 15 fractions and on one fraction for 5 fraction treatment regimens. Dose area statistics for area of skin receiving > 50% of the prescribed dose will be reported for each patient at the central coordinating centre, based on the average measured dose distribution for each patient.

#### *Radiation therapist *experience

A questionnaire specific to this study [see Additional file 2] will be completed by treating radiation therapists to assess their experience with breast positioning in both treatment arms regarding factors including ergonomic concerns, workflow, convenience, and perceived drawbacks. For each patient, the survey should be completed by two treating therapists during the last two weeks of treatment.

#### Workflow

Treatment and setup time will be measured weekly until treatment completion.

### Statistics and analysis

#### Sample size calculation and recruitment period

Based on evidence from previous studies [5–8], we expect a control arm rate of 50% for any degree of MD in the IMF. Due to a reduction in skin folds and reduction in V105% with reduced breast separation, we hypothesize a rate of 30% moist desquamation in the study arm (i.e., an absolute decrease in incidence of 20%). For this effect size with 80% power and two-tailed α = 0.05, the sample size is 93 patients in each arm. Because of the nature of the study and 10% observed loss to follow-up in our pilot study, we will enroll 110 patients in each arm.

We expect that a 3-year recruitment period will be sufficient for the trial. A similar multicentre study from our team [5] achieved a similar recruitment rate in the same patient population drawing from only two centres.

#### Randomization procedure and tracking

The random allocation lists will be computer-generated by a statistician not associated with the study. Randomization will use permuted blocks of random size. Stratification is by dose fractionation, by presence of IMF skin fold, and by centre.

The allocations will be uploaded into a password-protected website on secure hospital servers. When an eligible and consenting patient is identified in advance of their CT simulation appointment, the study coordinator or study radiation therapist will enter a patient identification code, and the computer will issue the allocation and a patient study number. An automated audit trail will track the patient identifier and study number, the treatment allocation, and the date, time, and site of the transaction.

#### *Statistical methods – *primary* outcome*

The proportion of patients experiencing MD in the IMF in the two arms will be compared using logistic regression to assess the effect of treatment, adjusting for brassiere cup size, skin fold size, BMI, smoking status, and dose fractionation schedule. An unadjusted comparison will also be made using the chi-square test, or Fisher’s exact test, if appropriate. Those participants who do not complete all follow-up but have reported MD are included in the analysis. Those who do not complete all follow-up and do not report MD will be excluded from the primary analysis. A sensitivity analysis including those lost to follow-up will be done using multiple imputations for missing data.

Baseline descriptive variables (e.g., age, smoking status, BMI, as described in the section Baseline Assessment) will be compared descriptively between the two arms of the study and assessed for imbalance. Formal statistical comparisons of baseline characteristics in RCTs are not advised [35].

#### Statistical methods – secondary outcomes

The eight secondary endpoints will be compared between the two study arms as follows:

- Degree of MD in the IMF: Peak measured area (cm^2^) of patchy MD and confluent MD will be compared where data is available. It is anticipated that a good proportion of patients will have no MD and hence a measured area of zero. Thus, we will divide the outcome into four categories, the first being zero and the other three will be equal sized groups based on their area score. We will then use the linear trend chi-square test to determine whether the groups differ in their distribution of scores.
- Peak (worst) modified NCI CTCAE scores for radiation dermatitis will be compared using the chi-square test for trend [36].
- Dose-volume statistics: values for heart, lung, and body will be compared using the Mann–Whitney U test.
- Dose-area statistics (*in-vivo* dosimetry): Measured skin dose-area statistics will be compared within both study arms. The measured skin dose-area data for patients developing MD in the location where the dose was measured will be compared with that of patients not developing MD in that location to determine if a dose-area metric predictive of MD can be found for either arm.
- PRO: The peak (worst) PRO breast symptom scores will be compared using a between-groups t-test, or if data do not conform to test assumptions, the non-parametric Mann–Whitney U test. The requirement for steroid cream and analgesics will be compared using the chi-square test, or Fisher’s exact test, if appropriate.
- Clinical workflow: Time for patient setup will be compared between study arms using a between groups t-test.
- Reproducibility of patient setup, as assessed by variation in surgical clip positions measured on imaging will be compared between study arms using the F-test.
- Therapist satisfaction: Therapist satisfaction with the setup procedure as measured by the questionnaires will be compared using a linear trend chi-squared test.

#### Interim analysis

A single interim analysis of efficacy will take place when half of the patients have completed follow-up. The trial will use the Peto approach [37] whereby the criterion for statistical significance at the interim analysis will be set at *p* < 0.001, and the criterion at the final analysis will be set at p < 0.05, which will maintain an overall α = 0.05. The Data Safety Monitoring Committee (DSMC) will use this guideline in combination with data on toxicity and secondary outcomes to decide whether to recommend continuation or termination of the trial.

Any deviations from the original statistical plans will be described and justified in an amendment to the protocol and/or in the final report, as appropriate.

### Safety assessment

The DSMC, consisting of one radiation oncologist, one medical physicist, and one biostatistician, will review data relating to safety and efficacy. The DSMC will conduct and review interim analyses and ensure the continued scientific validity and merit of the study. There will be one interim review conducted by the DSMC as described above.

Safety will be monitored through therapist daily interactions with the patient while on treatment, skin assessments, and PRO. NCI CTCAE V4.0 scores below grade 4 are expected in both arms. Adverse events (AEs) and severe adverse events (SAEs), as defined by the International Conference on Harmonization (ICH) [38], will be detected by the treating therapist at the time of treatment or follow-up assessment, or reported by the patient. Examples of adverse events (AEs) are skin reactions above grade 3, a treatment unit collision with the patient, or a patient fall during treatment setup. A PRO “yes” response to Q11 indicating the patient requires additional support to manage treatment related changes in skin will trigger a follow-up phone call to the patient to determine if an AE has occurred and provide the patient with appropriate management. The investigator and qualified designees will remain responsible for following up on AEs that are serious, considered related to the study intervention or study procedures, or that caused the participant to discontinue the study intervention. After the initial AE/SAE report, the investigator will proactively follow each participant at subsequent visits/contacts. All SAEs will be followed until resolution, stabilization, the event is otherwise explained, or the participant is lost to follow-up.

All SAEs and AEs will be collected from the start of intervention until the last follow-up visit. All SAEs will be recorded and reported to the sponsor or designee immediately, prior to the next scheduled treatment, or if treatment is complete, within 24 h. Any updates to SAE data will also be submitted to the sponsor within 24 h. The trial will be discontinued if a significantly higher rate of SAE is observed for the experimental arm versus the control arm, or upon recommendation by the DSMC at the time of the interim analysis.

For routine toxicity management, any dose interruptions or dose reductions in either arm (at the discretion of the radiation oncologist) will be monitored throughout the study. All interruptions or reductions will be documented in the patient’s treatment record and noted on the subsequent skin assessment form. Our standard institutional policies and procedures for pregnancy will be followed in this study.

### Confidentiality and data storage

Participants will be assigned a unique identifier by the study sponsor. Any participant records or datasets that are transferred to the sponsor will contain the identifier only; participant names (or other identifying information) will not be transferred. The participant will be informed that their personal study-related data will be used by the sponsor in accordance with local data protection laws. The level of disclosure will also be explained to the participant who will be required to give consent for their data to be used as described in the informed consent form (ICF). The participant will be informed that their medical records may be examined by Clinical Quality Assurance auditors or other authorized personnel appointed by the sponsor, by appropriate research ethics board (REB) members, or by inspectors from regulatory authorities.

All participant data relating to the study will be recorded on printed forms or in electronic format for upload into a restricted-access database housed on secure hospital servers (RedCap). Database entry will occur at the coordinating study site. For other sites, printed data forms should be scanned and emailed to the coordinating site within two weeks of the last assessment for each patient. Original copies will be retained at the treating centres. Digital copies of data collection forms, as well as any photographs taken for skin assessments, will be stored on a secure server accessible only to study staff. Database access will be restricted to the study monitor and any staff who will be entering data. The case report form (CRF) will be reviewed for accuracy and signed off by the study physicist and/or lead study therapist at the treating centre prior to data entry.

Source data for treatment planning, imaging, and setup will be retained in our institutional Aria™ Radiation Therapy clinical database, per standard of care for all patients. Source data for PRO data will be retained in the PRO database and may be uploaded into the RedCap database for analysis. A regular backup of the study databases will be performed.

Records and documents pertaining to the conduct of this study, including signed ICFs, will be retained by the investigator for 25 years after study completion. No records will be destroyed during the retention period or transferred to other locations or parties without the written approval of the sponsor. At the close of the study, the study coordinator will collect all study-related data from participating centres to be stored in a single location.

### Informed consent

Pre-screening for eligible patients who have an IMF skin fold and/or lateral ptosis in supine positioning will be done by the attending radiation oncologist or study therapist at the new patient assessment appointment. The study will be discussed at this appointment and participants will be informed that their participation is voluntary. The investigator or delegate will explain the nature of the study to the participant (or a legally authorized representative for the participant, as appropriate) and answer all questions regarding the study.

Voluntary, written, dated, and signed ICFs for participation in the study will be obtained prior to performing any study-related procedures. The radiation oncologist, study therapist, or study coordinator will provide ICFs to potential participants allowing adequate time for review prior to the CT simulation appointment. The ICF will meet the requirements of the REB and study center. The authorized person obtaining the informed consent must also sign and date the ICF. A copy of the ICF will be provided to the participant.

Screening evaluations will be completed and reviewed to confirm that participants meet all remaining eligibility criteria prior to enrolment. A screening log will be maintained to record details of all participants screened, to confirm eligibility, and to record reasons for screening failure, as applicable. The medical record will include a statement that written informed consent was obtained before the participant was enrolled in the study and the date the written consent was obtained.

Extension of this study to look at longer-term toxicities of breast RT including cosmesis, secondary cancer induction, recurrence, skin, lung, and heart toxicity is under consideration. Patients will be asked if they consent to having their data used in subsequent analysis.

### Participant withdrawal

A participant may withdraw from the study for any reason at any time. Patients may also be withdrawn from the study at the discretion of the investigator and/or treatment team, for example, if problems arise with healing of the surgical wound if this interferes with the required skin assessments or causes treatment interruption or delay. Participants withdrawn from the study shall continue to receive appropriate treatment as decided upon with their radiation oncologist. Data collected up to the point of withdrawal will remain in the study.

Patients in the control arm who withdraw from the study post-CT simulation will continue with treatment as planned, with no interruption. Patients in the experimental study arm who withdraw from the study will decide, in discussion with the treating oncologist, whether to continue with CARA or to change to the standard of care breast positioning method. In the latter case, the patient will be required to undergo repeat CT simulation and planning with the standard method. This process typically requires one week and may be done currently with continued treatment with CARA if desired. Otherwise, a treatment interruption may be required.

A participant is considered to have completed the study if they have completed all phases of the study including treatment and the final PRO questionnaire. If a participant fails to complete a follow-up PRO assessment, the site will attempt to contact the participant weekly and counsel the participant on the importance of maintaining the assigned follow-up schedule and ascertain whether the participant wishes to and/or should continue in the study. Should the participant remain unreachable out to eight weeks from the start of RT, they will be considered to have withdrawn from the study.

### Regulatory/ethical considerations

This study has been reviewed by an appropriate REB and will be conducted in accordance with applicable laws and regulations (e.g. Health Canada device regulations) and ICH-GCP guidelines.

The investigator will provide oversight of the study, including conduct and adherence to guidelines and regulations. Written summaries of the status of the study will be provided to the REB annually (or more frequently, as appropriate) and the REB will be notified of any SAEs, protocol violations, or significant safety findings.

Participating sites will be required to credential for this study by submitting treatment planning test cases and completing skin assessment training. A credentialing committee consisting of a least one physicist and one therapist will review submitted data and confirm when the site is approved. Upon study completion, closure of study sites will include collecting all required documents and supplies and a study-site closure visit. Early study-site closure may be initiated at any time, for reasons including failure to comply with protocols, the REB, ICH-GCP guidelines, local health authorities, or inadequate recruitment of participants.

The results of this study may be published or presented at scientific meetings. Investigators agree to submit all manuscripts or abstracts to the sponsor before submission. This allows the sponsor to protect proprietary information and to provide comments. The sponsor will comply with the requirements for publication of study results. In accordance with standard practice, the sponsor will generally support publication of multicenter studies only in their entirety and not as individual site data. In this case, a coordinating investigator will be designated by mutual agreement. Authorship will be determined by mutual agreement and in line with International Committee of Medical Journal Editors authorship requirements.

## Discussion

There is an unmet need for improved supine breast positioning for patients with large or pendulous breasts undergoing RT. MD in the infra-mammary area is common in this patient population and is associated with decreased quality of life. The primary objective of this study is to determine if positioning with the CARA V3.0 device will reduce the incidence of MD in the IMF compared to the current standard of care. After several preliminary and pilot studies, this is the first RCT to measure the effectiveness of CARA to reduce MD. It is expected that the IMF elimination reduce acute toxicity of RT in this patient population. Several dose-volume and dose-area parameters will be measured to fully appreciate the potential of the CARA intervention. A limitation of this study is that it is focused on acute toxicity measurement and longer-term lung and heart toxicities are not currently included. Future investigation of these endpoints is under consideration. A strength of the study is that we will be measuring clinical workflow factors including setup time, reproducibility, radiation therapist experience, and patient experience to understand factors that may influence the integration of CARA into standard breast RT protocols.

## Supplementary Information

**Additional file 1. **CARA PRO Questionnaire. Survey distributed to patients for collection ofpatient-reported outcomes throughout the study.

**Additional file 2.** CARA RT Experience Survey. Survey distributed toradiation therapists to measure variables including ease of setup, ergonomics,and overall experience using CARA throughout the study.

## Data Availability

Not applicable.

## Abbreviations

AE: Adverse Event
BMI: Body Mass Index
CARA: Carbon-Fibre Adjustable Reusable Accessory
CBCT: Cone Beam CT
CT: Computed Tomography
CTCAE: Common Terminology Criteria for Adverse Events
CTV: Clinical Target Volume
DSMC: Data Safety Monitoring Committee
Gy: Gray
ICF: Informed Consent Form
ICH-GCP: International Council for Harmonization—Good Clinical Practice
IMF: Infra-Mammary Fold
IMRT: Intensity Modulated RT
kV: Kilovolt
MD: Moist Desquamation
MV: Megavolt
NCI: National Cancer Institute
OAR: Organ At Risk
OTV: Optimization Target Volume
POSI: Prospective Outcomes and Surveillance Initiative
PTV: Planning Target Volume
PRO: Patient-Reported Outcome
RCT: Randomized Controlled Trial
REB: Research Ethics Board
RT: Radiation Therapy
RTOG: Radiation Therapy Oncology Group
SAE: Serious Adverse Event
V20Gy: Volume of an organ receiving a dose of 20 Gy or more
V105%: Volume of an organ receiving 105% or more of the prescribed dose
