# Supplementary material for: A novel carbon-fibre adjustable reusable accessory (CARA) for supine breast positioning to reduce toxicity in breast adjuvant radiotherapy: a study protocol for a multicentre phase III randomized controlled trial
Source: BMC Cancer. 2022 Jun 20;22:673. doi: 10.1186/s12885-022-09759-y (PMC9208179; doi:10.1186/s12885-022-09759-y)
Supplement: Supplementary file 1 — Additional file 1. CARA PRO Questionnaire. Survey distributed to patients for collection ofpatient-reported outcomes throughout the study. [file 12885_2022_9759_MOESM1_ESM.pdf]

# Prospective Outcomes and Support Initiative (POSI)
# PRO Questionnaire for the CARA RCT Study

Date: ______________ (dd/mm/yy)     BCCA#: ___________________

*Please circle or mark one number per line to indicate your response.*

**Over the past 7 days:**

**1. Have you used any skin care products in your breast area?**
*(select none or all that apply)*

None
- - - - - - - - - - - - - - - - - - - - - - - - - - - - - - - - - - - -

- ☐ Regular moisturizer (eg: Glaxol®, Lubriderm®, Aveeno®, etc.)
- ☐ Steroid cream ( eg: betamethasone or hydrocortisone)
- ☐ Saline soaks
- ☐ Antibiotic cream (eg: Flamazine® or Polysporin®)
- ☐ Other not listed above

| | | Not At All | A Little bit | Quite a Bit | Very Much |
|---|---|---|---|---|---|
| **2.** | **Have you felt tired?** | 0 | 1 | 2 | 3 |
| **3.** | **Did you have pain or tenderness in your breast or chest area?** | 0 | 1 | 2 | 3 |
| | *if you had pain or tenderness* **3a. Have you taken medication for the pain in your breast or chest area?** | 0 | 1 | 2 | 3 |

# PRO Questionnaire for the CARA RCT Study

Date: ______________ (dd/mm/yy)  BCCA#: ___________________

*If you have started or completed your radiation treatments, please complete the remaining questions:*

| Over the past 7 days, in the radiation treatment area: | | Not At All | A Little bit | Quite a Bit | Very Much |
|---|---|---|---|---|---|
| 4. | Has the skin on your breast or chest area been red or darker than usual? | 0 | 1 | 2 | 3 |
| 5. | Has the skin on your breast or chest area been itchy? | 0 | 1 | 2 | 3 |
| 6. | Has the skin on your breast or chest area been dry or flaky? | 0 | 1 | 2 | 3 |
| 7. | a) Do you have open skin on your breast or chest? | Yes or No | | | |
| (If answered Yes to 7a) | | | | | |
| | b) Is there open skin in the fold under your breast? | 0 | 1 | 2 | 3 |
| | c) Is there open skin near your armpit area? | 0 | 1 | 2 | 3 |
| | d) Is there open skin near your breastbone/ sternum? | 0 | 1 | 2 | 3 |
| | e) Is there open skin near the top of your chest/collarbone? | 0 | 1 | 2 | 3 |
| | f) Is there open skin in your nipple area? | 0 | 1 | 2 | 3 |
| | g) Is your open skin painful? | 0 | 1 | 2 | 3 |
| | h) Is your open skin bothersome? | 0 | 1 | 2 | 3 |

## Prospective Outcomes and Support Initiative (POSI)
## PRO Questionnaire for the CARA RCT Study

Date: ______________ (dd/mm/yy) BCCA#: ___________________

| | | Not At All | A Little bit | Quite a Bit | Very Much |
|---|---|---|---|---|---|
| **8.** | Have you experienced discomfort in your breast while being positioned for your radiation treatment? | **0** | **1** | **2** | **3** |
| **9.** | Have the changes in your skin as a result of your breast cancer treatment interfered with your ability to do regular activities such as work outside or inside the home, social or recreational activities? | **0** | **1** | **2** | **3** |
| **10.** | Have the changes in your skin as a result of your breast cancer treatment interfered with your sleep? | **0** | **1** | **2** | **3** |
| **11.** | Do you have access to the support you need to manage the changes in your skin occurring as a result of treatment? | **Yes or No** | | | |

*Please include any comments you wish to share with us:*

_______________________________________________

_______________________________________________

_______________________________________________

_______________________________________________

_______________________________________________

*Thank-you for completing this questionnaire*
