# Supplementary material for: A novel carbon-fibre adjustable reusable accessory (CARA) for supine breast positioning to reduce toxicity in breast adjuvant radiotherapy: a study protocol for a multicentre phase III randomized controlled trial
Source: BMC Cancer. 2022 Jun 20;22:673. doi: 10.1186/s12885-022-09759-y (PMC9208179; doi:10.1186/s12885-022-09759-y)
Supplement: Supplementary file 2 — Additional file 2. CARA RT Experience Survey. Survey distributed toradiation therapists to measure variables including ease of setup, ergonomics,and overall experience using CARA throughout the study. [file 12885_2022_9759_MOESM2_ESM.pdf]

RT Experience Survey for the CARA RCT Study

Date: ____________ (dd/mm/yy) **Unit:** _______________ **RT Initials**: ___________

Patient Study ID:____________ Patient Name:___________________

Please mark one number per line to indicate your response.

**Over the course of treating this patient:**	**Not at all**	**Somewhat**	**Mostly**	**Very Much**
	**1**	**2**	**3**	**4**
**1. Was the setup reproducible?**				
**2. Was the setup easy to accomplish?**				
**3. How satisfied were you with the setup?**				
**4. Did you experience any ergonomic concerns during this patient's setup? If yes, please describe below.**		**Yes / No**		
**5. Do you have any concerns about the setup method used? If yes, please provide detail below.**		**Yes / No**		

Comments:

____

____

____

____

____

____.
